# Crohn’s disease but not ulcerative colitis elevated risk of end-stage renal disease and mortality: A Taiwan retrospective cohort study

**DOI:** 10.1097/MD.0000000000042026

**Published:** 2025-04-04

**Authors:** Ming-Che Chuang, Tzu-Ju Hsu, Fuu-Jen Tsai, Jye-Lin Hsu, Tsung-Yu Tsai

**Affiliations:** a Department of Internal Medicine, Center for Digestive Medicine, China Medical University Hospital, Taichung, Taiwan; b Management Office for Health Data, Clinical Trial Research Center, China Medical University Hospital, Taichung, Taiwan; c Department of Statistics, M.A., Master’s Program in Statistics and Actuarial Science, Feng Chia University, Taichung, Taiwan; d School of Chinese Medicine, College of Chinese Medicine, China Medical University, Taichung, Taiwan; e Department of Medical Research, China Medical University Hospital, Taichung, Taiwan; f Division of Medical Genetics, China Medical University Children’s Hospital, Taichung, Taiwan; g Department of Biotechnology and Bioinformatics, Asia University, Taichung, Taiwan; h Graduate Institute of Biomedical Sciences, China Medical University, Taichung, Taiwan; i Drug Development Center, China Medical University, Taichung, Taiwan; j School of Medicine, China Medical University, Taichung, Taiwan; k Translational Cell Therapy Center, China Medical University Hospital, Taichung, Taiwan; l Immunology Research Center, National Health Research Institutes, Maioli, Taiwan.

**Keywords:** Crohn disease, end-stage renal disease, inflammatory bowel disease, mortality, ulcerative colitis

## Abstract

Inflammatory bowel disease (IBD) is an autoinflammatory disease which may affect extraintestinal organs, including kidney. However, rare research showed that patients with IBD have higher risk of end-stage renal disease (ESRD). Furthermore, lack of studies compared the potential risk of ESRD and mortality among patients with ulcerative colitis (UC) and Crohn disease (CD). We conducted a nationwide cohort study using the National Health Insurance database in Taiwan, from January 2008 to December 2018. A total of 3204 patients diagnosed with IBD were enrolled. IBD cases were identified through the presence of a catastrophic illness certificate, including CD and UC. The study outcomes were the incidence of ESRD and mortality. ESRD diagnosis required a serious illness certificate and was identified using the corresponding ICD-10-CM codes. Mortality was recorded in the Taiwan Death Registry linked with the National Health Insurance database, Cox proportional hazards models were used to estimate the risk factors for ESRD and mortality among IBD patients. CD patients had a significantly higher risk of ESRD (adjust hazard ratio: 2.32, 95% confidence interval: 1.28–4.18) and mortality (adjust hazard ratio: 1.80, 95% confidence interval: 1.37–2.35) compared to healthy individuals. UC patients showed no difference in the risk of ESRD compared to healthy individuals. Instead, among IBD patients, UC poses a relatively lower risk for ESRD compared to other factors like age and other comorbidities. Elevated risk of ESRD and mortality was only noted in patients with CD but not UC. Surprisingly, UC patients had lower risk of ESRD and mortality than CD patients. These findings highlight distinctive patterns of risk associated with CD and UC, emphasizing the importance of considering disease subtype when assessing outcomes such as ESRD and mortality.

## 1. Introduction

Inflammatory bowel disease (IBD), divided into Crohn disease (CD) and ulcerative colitis (UC), is an autoinflammatory disease affecting the gastrointestinal system, leading to symptoms such as diarrhea, bloody stools, and abdominal pain. Additionally, IBD presents extraintestinal manifestations (EIMs) with a prevalence of 6% to 47%, which can be categorized based on their relationship with intestinal inflammation. Some EIMs, such as peripheral arthritis, oral aphthous ulcers, episcleritis, and erythema nodosum, tend to parallel IBD activity and improve with treatment of the underlying gut inflammation. Others sometimes indicated as extraintestinal complications, including anterior uveitis, ankylosing spondylitis, and primary sclerosing cholangitis, follow a distinct course independent of intestinal disease activity.^[1,2]^ Emerging evidence suggests that renal involvement may also be considered an EIM in IBD. In a previous study, the predominant renal diseases observed in patients with IBD were nephrolithiasis, tubulointerstitial nephritis, glomerulonephritis, and amyloidosis.^[3]^ It is worth noting that the autoinflammatory mechanisms associated with IBD may contribute to an increased risk of renal involvement, such as glomerulonephritis, potentially leading to chronic kidney disease (CKD).^[4]^

IBD patients should be mindful of the risk of CKD. In a retrospective cohort study conducted by Ravy K. Vajravelu et al, it was observed that IBD patients may face an elevated risk of CKD, with this risk being inversely associated with age.^[5]^ Similarly, Mengyi Liu et al reported in a similar study that IBD patients, based on data from the UK data bank, had a higher risk of incident CKD and acute kidney injury, particularly in younger individuals.^[6]^ Furthermore, it is essential to note that CKD has the potential to progress to end-stage renal disease (ESRD). To date, there has been only 1 reported instance indicating an increased risk of ESRD in patients with CD, whereas there has not been a direct comparison of the risk of ESRD between individuals with CD and UC.^[7]^

Although the above studies have indicated that IBD increases the risk of CKD and ESRD, there remain certain gaps in understanding. Firstly, there is a scarcity of comparisons regarding the risk of CKD and ESRD between CD and UC. Secondly, the prognosis and CKD risk specifically associated with CD or UC are not well-established. Third, the above study was mostly done in UK and seldom have Asia data. In light of these unknowns, our objective is to comprehensively assess the risk of CKD, ESRD, and overall prognosis in patients with CD and UC. Our aim is to investigate the clinical incidence of renal involvement in IBD patients and provide insights into the comparative risks and outcomes associated with ESRD between CD and UC. This research aims to enhance our understanding of the renal implications of different IBD subtypes, especially in Asia.

## 2. Materials and methods

### 2.1. Data source

The National Health Insurance Research Database (NHIRD) came from the Data Science Center of the Statistics Department of the Taiwan Ministry of Health Services, which covered 99% of the national medical information. The data was close to the actual medical situation in Taiwan, so the research value was high. The disease codes in the database followed the 9th revision and 10th revision of the International Classification of Diseases. This study was approved by the Institutional Review Board of the Research Ethics Committee of China Medical University Hospital (CMUH110-REC3-133 (CR-2)).

### 2.2. Study population

The study interval was IBD patients diagnosed from 2008 to 2018 between the ages of 18 and 100. IBD patients were defined as those who had applied for a catastrophic illness certificate (HV), which could be divided into CD and UC. The ICD-9-CM code of CD and UC were 555, 556, and ICD-10M code were K50, K51. The index date of the case group was defined as the date of the first application for a catastrophic illness certificate, and the index date of the reference group was defined as a random date after January 1, 2008. “In Taiwan, the application for a catastrophic illness certificate requires pathological confirmation and colonoscopic imaging of IBD. As this certification reduces treatment costs, most IBD patients obtain it, minimizing selection bias.” Patients with IBD were matched 1:1 with non-IBD individuals using propensity score matching through the nearest neighbor method, ensuring comparability based on gender, age, monthly income (NTD), urbanization level, comorbidities, and the year of the index date. Initially, matching was performed to the 8th digit, and if no match was found, it was progressively relaxed to the first digit. The matching process began with a caliper width of 0.0000001, which was gradually increased to 0.1 for unmatched cases. To refine the matching, we reassessed the criteria and conducted a rematch using a greedy algorithm, ensuring that each IBD patient was paired with the closest propensity score match. The exclusion criteria for this study included: (1) missing gender information; (2) diagnosed with CKD or died before the index date; (3) diagnosed with related diseases (related diseases include segmental GN, membranous GN, MPGN, PSGN, diabetes mellitus (DM) nephropathy, hypertensive nephropathy, hereditary nephropathy, drug related nephropathy, cryoglobulinemia purpura, and thrombotic purpura); (4) diagnosed with CD and UC (Figure S1, Supplemental Digital Content, http://links.lww.com/MD/O607).

### 2.3. Outcome and covariate

The outcome was defined as patients diagnosed with ESRD disease or mortality. ESRD needed to apply for a serious illness certificate, and the ICD-10-CM code followed N18.5, N18.6, Z99.2. These codes indicate that the patients are currently undergoing regular dialysis. In addition, covariates include gender, age, monthly income, urbanization level, and comorbidities. The monthly income unit was NTD, urbanization levels were defined into 3 groups: high (metropolitan cities), medium (small cities and suburbs areas), and low (rural areas). Comorbidities were defined as at least 2 outpatient records or at least 1 hospitalization record, which include diabetes (ICD-9-CM: 250; ICD-10-CM: E08-E13), hypertension (ICD-9-CM: 401-405; ICD-10-CM: I10-I13, I15, N26.2), heart failure (ICD-9-CM: 428; ICD-10-CM: I50). Mortality in this study was defined as all-cause mortality, as recorded in the Taiwan Death Registry linked with the National Health Insurance database. This registry provides official records of the date and cause of death for each deceased individual. Under Taiwanese law, all death certificates must be completed by a physician, ensuring the accuracy and completeness of the mortality data.

### 2.4. Statistical analysis

Categorical variables and continuous variables were represented by number (percentage) and mean (standard deviation), and chi-square test and *t* tests were used to test whether there were differences between case and reference group, respectively. The incidence rate was the number of events divided by the total number of per 1000 person-years. The crude hazard ratios (HR) and the adjusted hazard ratios (aHR) were estimated by univariate and multivariate cox proportional hazards models, where adjustment variables were based on gender, age, monthly income, urbanization level, and comorbidities. The cumulative incidence curve between groups were estimated by the Kaplan–Meier method, and the difference between the curves was verified by the log rank test. If the *P*-value on 2 sides of this study was <.05, the result was statistically significant. All statistical analyses were performed by SAS software, version 9.4 (SAS Institute Inc., Cary, NC). The graph was performed by RStudio.

## 3. Results

We totally enrolled 3204 patients, UC 2311 patients and CD 893 patients. The baseline characteristics of IBD and reference group were matched according to gender, age, monthly income, urbanization level, comorbidity, and the year of index date by propensity score matching at a ratio of 1:1, as shown in Table 1. Men accounted for more than half of the total in both case and reference group, with an average (standard deviation) age of 44.27 (±15.18) years and 44.64 (±16.68) years, respectively. The monthly income of NT$20,000 to 40,000 (51.03% vs 53.09%) accounted for the majority of the population.

**Table 1:** Baseline characteristics of inflammatory bowel disease cohort and control cohort.

Variables	IBD				*P*-value
	No (N = 3204)		Yes (N = 3204)		
	n	%	n	%	
IBD					
Crohn disease	–	–	893	27.87	
Ulcerative colitis	–	–	2311	72.13	
Gender					
Female	1069	33.36	1148	35.83	.04
Male	2135	66.64	2056	64.17	
Age, years					
18–40	1367	42.67	1385	43.23	<.001
41–60	1174	36.64	1299	40.54	
≥60	663	20.69	520	16.23	
Mean ± SD*	44.64	16.68	44.27	15.18	.36
Monthly income (NTD)					
<20,000	566	17.67	573	17.88	.21
20,000–40,000	1701	53.09	1635	51.03	
>40,000	937	29.24	996	31.09	
Urbanization level					
High	1350	42.13	1236	38.58	<.001
Medium	674	21.04	603	18.82	
Low	1180	36.83	1365	42.60	
Comorbidities					
Diabetes	521	16.26	241	7.52	<.001
Hypertension	778	24.28	529	16.51	<.001
Heart failure	409	12.77	40	1.25	<.001
Follow-up time, years, mean ± SD*					
End-stage renal disease	5.21	3.11	5.41	3.23	.01
Mortality	5.26	3.12	5.46	3.24	.01

IBD = inflammatory bowel disease, NTD = New Taiwan Dollars, SD = standard deviation.

* *t* test.

### 3.1. CD patients had higher risk of ESRD and mortality compared to healthy individuals

In our investigation into the risk of ESRD and mortality among IBD patients, we evaluated the HR for these outcomes in our study population. There were no statistically significant for the risk of ESRD and mortality in IBD patients compared to healthy individuals (Table 2a). But if we focus on patients with CD, we found a significantly increased risk of ESRD and mortality compared to healthy individuals (ESRD: adjusted HR = 2.32, 95% confidence interval [CI] = 1.28–4.18, *P* = .0054; mortality: adjusted HR = 1.80, 95% CI = 1.37–2.35 *P* < .001) (Table 2b).

**Table 2:** Cox proportional hazards regression analysis for the risk of end-stage renal disease and mortality.

(a) Cox proportional hazards regression analysis for the risk of end-stage renal disease and mortality by inflammatory bowel disease.																
Variables	IBD										cHR	(95% CI)	*P*-value	aHR†	(95% CI)	*P*-value
	No						Yes									
	Event		PY	IR			Event		PY	IR						
End-stage renal disease	52		16,706	3.11			38		17,336	2.19	0.70	(0.46, 1.06)	.0891	1.36	(0.85, 2.20)	.20
Mortality	256		16,852	15.19			166		17,491	9.49	0.63	(0.52, 0.76)***	<.001	0.93	(0.75, 1.15)	.51

(b) Cox proportional hazards regression analysis for the risk of end-stage renal disease and mortality by Crhon disease.																
Variables	Crohn disease										cHR	(95% CI)	*P*-value	aHR†	(95% CI)	*P*-value
	No						Yes									
	Event		PY	IR			Event		PY	IR						
End-stage renal disease	76		29,769	2.55			14		4273	3.28	1.31	(0.74, 2.33)	.3475	2.32	(1.28, 4.18)**	.0054
Mortality	357		30,025	11.89			65		4317	15.06	1.25	(0.96, 1.63)	.0931	1.80	(1.37, 2.35)***	<.001

(c) Cox proportional hazards regression analysis for the risk of end-stage renal disease and mortality by ulcerative colitis.																
Variables	Ulcerative colitis										cHR	(95% CI)	*P*-value	aHR†	(95% CI)	*P*-value
	No					Yes										
	Event	PY			IR	Event		PY		IR						
End-stage renal disease	66	20,979			3.15	24		13,063		1.84	0.57	(0.36, 0.91)*	.0184	0.88	(0.53, 1.46)	.63
Mortality	321	21,169			15.16	101		13,173		7.67	0.51	(0.41, 0.64)***	<.001	0.66	(0.52, 0.83)***	<.001

aHR = adjusted hazard ratio, cHR = crude hazard ratio, IBD = Inflammatory Bowel disease, IR = incidence rate, per 1000 person-years, PY = person-year.

† Adjusted HR estimated by the model including the variables of gender, age, monthly income, urbanization level, and comorbidities.

* *P* < .05.

** *P* < .01.

*** *P* < .001.

### 3.2. UC patients had lower risk of mortality compared to healthy individuals

We further evaluated the risk of ESRD and mortality in patients with UC and estimated the HR of outcomes. Compared with patients without UC, the risk of mortality in patients with UC was significantly reduced (adjusted HR = 0.66, 95% CI = 0.52–0.83, *P* < .001). However, there was not significant with the risk of ESRD in UC patients compared to healthy controls (*P* = .63) (Table 2c). The risk of ESRD and mortality was totally different between CD and UC.

### 3.3. CD patients had higher risk of ESRD and mortality compared to UC

**Figure 1.**
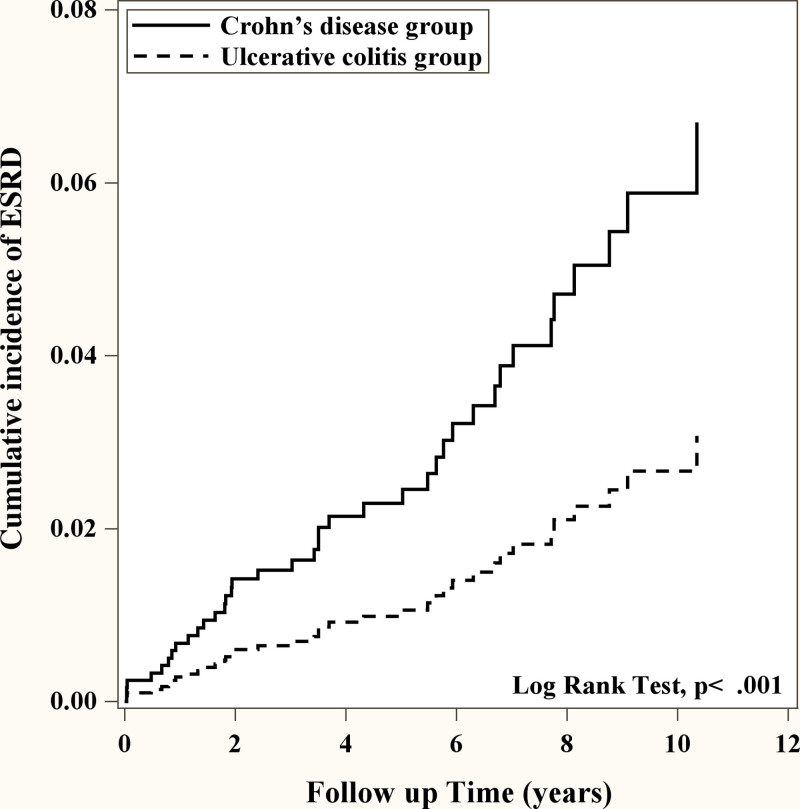
Kaplan–Meier curves of the cumulative incidence of ESRD during the follow-up period among patients with IBD subgroup. ESRD = end-stage renal disease, IBD = inflammatory bowel disease.

**Figure 2.**
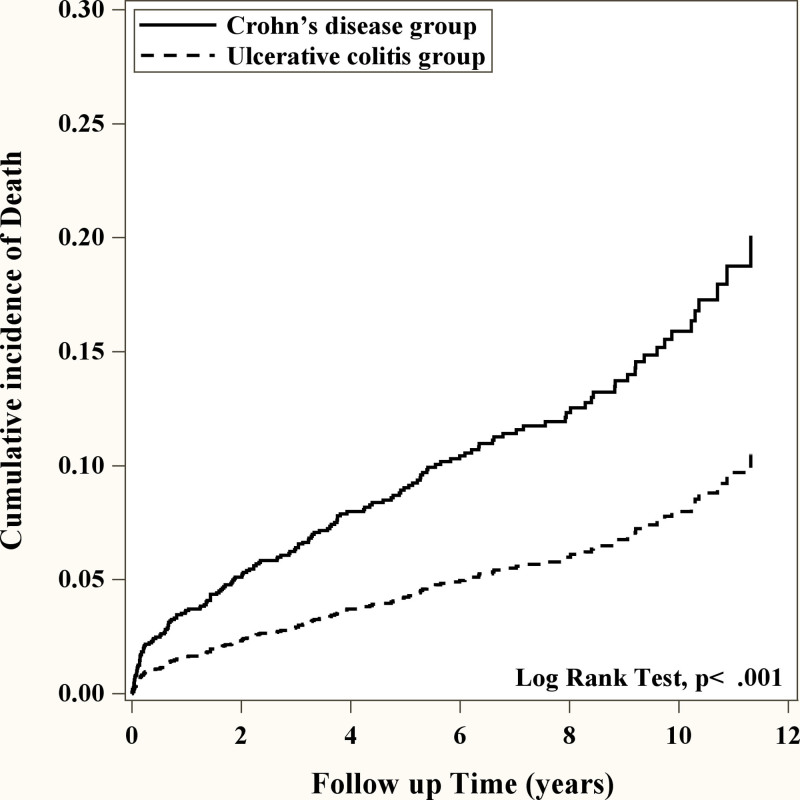
Kaplan–Meier curves of the cumulative incidence of mortality during the follow-up period among patients with IBD subgroup. IBD = inflammatory bowel disease.

Given the observed differences in the trends of risk for ESRD and mortality between UC and CD when compared to healthy controls, our focus is now on directly comparing these risks between UC and CD subgroups. Compared with CD, we found that the risk of ESRD was significantly decreased in patients diagnosed with UC (adjusted HR = 0.42, 95% CI = 0.21–0.81 *P* = .01) (Table 3). The cumulative incidence of ESRD in CD group was significantly higher than that in UC group (Fig. 1). More interestingly, in the age group compared with the age group 18 to 40 years old, 41 to 60, and ≥60 years old patients had a significantly increased risk of ESRD (41–60: adjusted HR = 1.95, 95% CI = 1.03–3.67, *P* = .04; ≥60: adjusted HR = 3.01, 95% CI = 1.53–5.92, *P* = .0014) (Table 3). We further investigated the relationship of comorbidity in our study group, the risk of ESRD with comorbidity was also significantly increased compared with those without comorbidity. (diabetes: adjusted HR = 2.40, 95% CI = 1.52–3.80, *P* < .001; hypertension: adjusted HR = 2.36, 95% CI = 1.45–3.84, *P* < .001; heart failure: adjusted HR = 3.29, 95% CI = 2.02–5.34, *P* < .001) (Table 3). The results suggest that, among IBD patients, UC poses a relatively lower risk for ESRD compared to factors like age and other comorbidities. We further evaluate the mortality of different types of IBD patients with ESRD. We found that the risk of mortality of in UC patients was significantly reduced compared to CD patients (adjusted HR = 0.43, 95% CI = 0.31–0.59, *P* < .001) (Table 4). The cumulative incidence of mortality in CD group was significantly higher than that in UC group (Fig. 2). More interestingly, the risk of mortality in men is significantly higher compared to women (adjusted HR = 1.32, 95% CI = 1.08–1.62, *P* = .0069) (Table 4). Similar to the result of ESRD, age more than 41 years old patients had a significantly increased risk of mortality compared to younger age (41–60: adjusted HR = 2.30, 95% CI = 1.68–3.14, *P* < .001; ≥60: adjusted HR = 6.20, 95% CI = 4.52–8.52, *P* < .001) (Table 4). Patients with comorbidity had significantly higher risk of mortality compared to those without comorbidity (diabetes: adjusted HR = 1.47, 95% CI = 1.18–1.83, *P* < .001; hypertension: adjusted HR = 1.31, 95% CI = 1.05–1.64, *P* = .02; heart failure: adjusted HR = 2.59, 95% CI = 2.04–3.29, *P* < .001) (Table 4). UC showed a relatively lower risk factor for mortality in IBD patients when compared to factors such as age, sex, and other comorbidities.

**Table 3:** Incidences and hazard ratios of end-stage renal disease by age, gender, and comorbidities.

Variables	End-stage renal disease			cHR	(95% CI)	*P*-value	aHR†	(95% CI)	*P*-value
	Event	PY	IR						
IBD subtype									
Crohn disease	14	4273	3.28	1.00	(reference)	-	1.00	(reference)	-
Ulcerative colitis	24	13,063	1.84	0.55	(0.28, 1.06)	.0727	0.42	(0.21, 0.81)*	.01
Gender									
Female	35	12,087	2.9	1.00	(reference)	-	1.00	(reference)	-
Male	55	21,955	2.51	0.88	(0.57, 1.34)	.5416	1.16	(0.75, 1.78)	.50
Age, year									
18–40	14	15,221	0.92	1.00	(reference)	-	1.00	(reference)	-
41–60	37	13,422	2.76	2.99	(1.62, 5.54)***	<.001	1.95	(1.03, 3.67)*	.04
≥60	39	5399	7.22	8.14	(4.41, 15.01)***	<.001	3.01	(1.53, 5.92)**	.0014
Comorbidities									
Diabetes									
No	49	29,811	1.64	1.00	(reference)	-	1.00	(reference)	-
Yes	41	4231	9.69	5.92	(3.91, 8.96)***	<.001	2.40	(1.52, 3.80)***	<.001
Hypertension									
No	36	26,944	1.34	1.00	(reference)	-	1.00	(reference)	-
Yes	54	7098	7.61	5.67	(3.72, 8.64)***	<.001	2.36	(1.45, 3.84)***	<.001
Heart failure									
No	57	31,694	1.80	1.00	(reference)	-	1.00	(reference)	-
Yes	33	2348	14.05	7.91	(5.15, 12.15)***	<.001	3.29	(2.02, 5.34)***	<.001

cHRaHR = adjusted hazard ratio, IBD = Inflammatory Bowel disease, IR = incidence rate, per 1000 person-years, PY = person-year.

† Adjusted HR estimated by the model including the variables of gender, age, monthly income, urbanization level, and comorbidities.

* *P* < .05.

** *P* < .01.

*** *P* < .001.

**Table 4:** Incidences and hazard ratios of mortality by age, gender, and comorbidities.

Variables	Mortality			cHR	(95% CI)	*P*-value	aHR†	(95% CI)	*P*-value
	Event	PY	IR						
IBD subtype									
Crohn disease	65	4317	15.06	1.00	(reference)	–	1.00	(reference)	-
Ulcerative colitis	101	13,173	7.67	0.52	(0.38, 0.71)***	<.001	0.43	(0.31, 0.59)***	<.001
Gender									
Female	143	12,177	11.74	1.00	(reference)	–	1.00	(reference)	-
Male	279	22,166	12.59	1.07	(0.87, 1.31)	.5215	1.32	(1.08, 1.62)**	.0069
Age, year									
18–40	57	15,265	3.73	1.00	(reference)	–	1.00	(reference)	-
41–60	141	13,564	10.40	2.78	(2.05, 3.79)***	<.001	2.30	(1.68, 3.14)***	<.001
≥60	224	5514	40.62	10.80	(8.09, 14.49)***	<.001	6.20	(4.52, 8.52)***	<.001
Comorbidities									
Diabetes									
No	284	29,998	9.47	1.00	(reference)	–	1.00	(reference)	-
Yes	138	4344	31.77	3.39	(2.76, 4.15)***	<.001	1.47	(1.18, 1.83)***	<.001
Hypertension									
No	217	27,079	8.01	1.00	(reference)	–	1.00	(reference)	-
Yes	205	7264	28.22	3.54	(2.93, 4.29)***	<.001	1.31	(1.05, 1.64)*	.02
Heart failure									
No	303	31,917	9.49	1.00	(reference)	–	1.00	(reference)	-
Yes	119	2425	49.07	5.19	(4.20, 6.42)***	<.001	2.59	(2.04, 3.29)***	<.001

aHR = adjusted hazard ratio, cHR = crude hazard ratio, IBD = inflammatory bowel disease, IR = incidence rate, per 1000 person-years, PY = person-year.

† Adjusted HR estimated by the model including the variables of gender, age, monthly income, urbanization level, and comorbidities.

* *P* < .05.

** *P* < .01.

*** *P* < .001.

## 4. Discussion

In the previous studies, it was observed that the prevalence of UC was higher than that of CD. Additionally, there was an imbalance in gender distribution, with a higher proportion of men compared to women.^[8–10]^ We got similar results in our study. In our data base, 64.2% were male and 72.1% were UC. Notably, our study also found that male IBD patients had a higher risk of mortality compared to females. This disparity may be influenced by factors such as the higher prevalence of smoking among males and the potential effects of hormones on the brain–gut microbiota axis, both of which could contribute to sex-based differences in IBD prognosis.^[11,12]^ Further research is needed to explore these associations. These demographic variations indicate the importance of considering both disease subtype and gender as key factors influencing IBD outcomes, particularly in the context of ESRD and mortality.

Several studies have indicated a reciprocal relationship between a diminished estimated glomerular filtration rate and the likelihood of developing IBD.^[13]^ In this study, we found that CD patients had higher risk of ESRD and with the prognosis of mortality, while UC patients had relatively lower risk in those. This finding pointed out the possible different of gut–kidney axis between UC and CD, which will be mentioned in the following paragraph. Besides, unlike other studies which showed the relative HR of ESRD caused by IBD declined with increasing age,^[5,6]^ our study found that the relative hazard of ESRD increased with age. The different results may be due to the fact that both of those studies’ endpoint focus on CKD, but our studies focus on ESRD. Therefore, to sum up both their and our study, although CKD happens early in IBD patients, they may progress to ESRD lately, according to their IBD type. The different results may be attributed to the fact that the mentioned studies primarily focused on CKD, whereas our study focused on ESRD. In summarizing findings from both sets of research, it appears that while CKD tends to occur early in patients with IBD, the progression to ESRD may occur later, and this progression may vary based on the type of IBD.

The prevalence of ESRD-related comorbidity in IBD patients was relatively low compared to non-IBD groups. In our study, patients with IBD have lower prevalence of DM (7.52% vs 16.26%), hypertension (16.51% vs 24.28%), and heart failure (1.25% vs 12.77%) than patients without IBD (Tables 1 and 5). In other studies, similar results were found. Kang EA et al reported that patients with IBD have lower association with hypertension (16.1% vs 18.3%) compared with those without IBD.^[14]^ Zheng et al also reported that patient with CD and UC have lower prevalence of DM (CD 16.4% vs 20%; UC 19.8% vs 22.8%), hypertension (CD 29.1% vs 33.5%; UC 33% vs 35.9%), and heart failure (CD 10.2% vs 11.9%; UC 12.4% vs 14.9%) compared with those without IBD respectively.^[15]^ These findings suggest that, despite having a lower prevalence of ESRD-related comorbidities, CD patients face a heightened risk of both ESRD and mortality when compared to those UC patients and healthy individuals. This disparity implies the existence of potentially distinct immune-related pathways in CD that impact the kidneys, pathways that have yet to be fully understood or explored.

**Table 5:** Baseline characteristics of inflammatory bowel disease subgroups.

Variables	IBD				
	No (N = 3204)		Yes (N = 3204)		
	n	%	n		%
IBD subtype					
Crhon disease			893		27.87
Ulcerative colitis			2311		72.13
Comorbidities					
Diabetes	521	16.26	241		7.52
Hypertension	778	24.28	529		16.51
Heart failure	409	12.77	40		1.25

Variables	UC				
			Yes (N = 2311)		
		n	%	
Comorbidities					
Diabetes			182	7.88	
Hypertension			408	17.65	
Heart failure			29	1.25	

Variables	CD				
			Yes (N = 893)		
			n	%	
Comorbidities					
Diabetes			59	6.61	
Hypertension			121	13.55	
Heart failure			11	1.23	

CD = Crohn disease, IBD = inflammatory bowel disease, UC = ulcerative colitis.

Several mechanisms affect the gut–kidney axis. The axis involves a complex interplay of immune responses, microbial activity, and various signaling molecules, emphasizing the importance of considering both the gut and the kidneys as integral components of systemic health.^[16–18]^ In clinical evidence, IgA nephropathy is the most common kidney biopsy diagnosis in patients with CD.^[19]^ Meanwhile, histological findings of renal biopsies in patients with IgA nephropathy and CD showed more severe glomerular sclerosis, interstitial fibrosis and tubular atrophy than those with IgA nephropathy without CD. Besides patients with IgA nephropathy and CD had more highly resistant clinical response to steroid treatment than patients with IgA nephropathy without CD.^[20]^ Moreover, patients with CD easily developed secondary amyloidosis than patient with those with UC.^[21]^ These findings emphasize the hint and need to explore the gut–kidney axis for comprehensive insights into the complex relationships between CD and renal outcomes.

Our study possesses several features. Firstly, like other studies using National Health Insurance Database, the identification of disease outcome relied on data reports. To improve the accuracy and dependability of diagnoses, we deliberately selected individuals holding certificates for catastrophic illnesses (HV) and with recurrent coding, thereby efficiently reducing potential biases. Furthermore, our research, characterized by a significant sample size, resulted in robust statistical power and alleviated selection bias. In addition, the outcomes of our study provide broad applicability within Taiwan, as the National Insurance Program extends coverage to over 99% of the nation’s residents. Nevertheless, it remains a possibility that patients who did not report symptoms or signs of UC, CD, or ESRD, and did not seek medical attention, could have gone undiagnosed. Consequently, there is a potential for misclassification of such individuals as control subjects in our study.

This study has several limitations. First, we lacked data on medication history and IBD-related surgical history, which are important factors given that some studies have suggested that biological therapy and colectomy may increase the risk of renal events and ESRD.^[22,23]^ Second, this study is subject to residual confounding due to the lack of data on smoking, lipid profiles, dietary habits, alcohol consumption, and disease severity. Smoking has a dual effect on IBD outcomes: while it is known to reduce the risk of UC and hospitalization rates, it also increases the risks of cancer and mortality,^[24]^ lack of smoking data in our study means we could not adjust for its potential protective effect against UC. Serum lipids in IBD patients are typically lower than in healthy individuals and correlate with disease severity, potentially serving as an indirect marker of inflammation.^[25]^ Without lipid data, we may have underestimated the role of metabolic disturbances in IBD-related kidney disease, as lower lipid levels could serve as an indirect marker of more severe inflammation. Alcohol effects on IBD vary by subtype and frequency and are generally not recommended.^[26]^ However, given that alcohol is generally not recommended for IBD patients, its exclusion may have had a limited impact on our findings. Additionally, the absence of disease severity classification in our dataset may have led to underestimation of the impact of aggressive IBD on renal outcomes. Lacking the above data may affect the study results.

We propose that the most 2 important residual confounding are smoking and serum lipids levels. While smoking has a protective effect against UC,^[24]^ it increases the risk of renal failure.^[27]^ IBD patients usually have lower lipid levels,^[25]^ while hypertriglyceridemia is a risk factor for chronic renal disease.^[28]^ These factors conflict with the risk factors for IBD and renal failure. We suggest future research to investigate the risk of ESRD and mortality in IBD patients with these variables.

## 5. Conclusion

In summary, the presented data indicates that individuals with CD face a notably elevated risk of ESRD and mortality in comparison to non-CD controls. Conversely, those with UC exhibit a lower risk of mortality compared to non-UC controls. Additionally, when comparing CD and UC patients directly, individuals with CD still have a significantly increased risk of both ESRD and mortality compared to those with UC. Moreover, UC is a relative lower risk factor of ESRD and mortality in IBD patients compared to other comorbidities. These findings highlight distinctive patterns of risk associated with CD and UC, emphasizing the importance of considering disease subtype when assessing outcomes such as ESRD and mortality.

## Author contributions

**Conceptualization:** Ming-Che Chuang, Jye-Lin Hsu, Tsung-Yu Tsai.

**Data curation:** Tzu-Ju Hsu, Fuu-Jen Tsai.

**Formal analysis:** Ming-Che Chuang.

**Funding acquisition:** Tsung-Yu Tsai.

**Methodology:** Tzu-Ju Hsu, Fuu-Jen Tsai.

**Supervision:** Tsung-Yu Tsai.

**Validation:** Tzu-Ju Hsu.

**Visualization:** Fuu-Jen Tsai, Tsung-Yu Tsai.

**Writing – original draft:** Ming-Che Chuang.

**Writing – review & editing:** Tsung-Yu Tsai.

## Supplementary Material


